# A Novel Segment-Based Approach for Improving Classification Performance of Transport Mode Detection

**DOI:** 10.3390/s18010087

**Published:** 2017-12-30

**Authors:** M. Amac Guvensan, Burak Dusun, Baris Can, H. Irem Turkmen

**Affiliations:** Department of Computer Engineering, Yildiz Technical University, 34220 Istanbul, Turkey; b.dusun@gmail.com (B.D.); bc.brscn@gmail.com (B.C.); irem@ce.yildiz.edu.tr (H.I.T.)

**Keywords:** transport mode detection, post-processing, smartphone, accelerometer, gyroscope, magnetometer, correction of misclassified vehicle types, pedestrian and vehicular activities

## Abstract

Transportation planning and solutions have an enormous impact on city life. To minimize the transport duration, urban planners should understand and elaborate the mobility of a city. Thus, researchers look toward monitoring people’s daily activities including transportation types and duration by taking advantage of individual’s smartphones. This paper introduces a novel segment-based transport mode detection architecture in order to improve the results of traditional classification algorithms in the literature. The proposed post-processing algorithm, namely the Healing algorithm, aims to correct the misclassification results of machine learning-based solutions. Our real-life test results show that the Healing algorithm could achieve up to 40% improvement of the classification results. As a result, the implemented mobile application could predict eight classes including stationary, walking, car, bus, tram, train, metro and ferry with a success rate of 95% thanks to the proposed multi-tier architecture and Healing algorithm.

## 1. Introduction

With the help of smartphones and IoT devices, the ability to monitor the environment and daily human activities has substantially increased from the beginning of the 2000s. Thus, activity recognition, especially on smartphones and smart watches, has became an important research area for over a decade.

Numerous promising applications such as smart home applications, healthcare monitoring solutions, security/surveillance systems and teleimmersion applications [1] require understanding the present activity of a person. In the literature, first, the researchers worked on the recognition of simple daily activities such as walking, running, climbing up/down, sitting, standing and biking. Later, researchers and urban planners realized that monitoring and analyzing the mobility with respect to city life would help to build smart city applications and implement intelligent transportation systems.

This paper focuses on a particular application area of activity recognition, namely transport mode detection, which is highly related to Intelligent Transportation Systems (ITS). The proliferation of mobile phones has allowed sensors to be portable enough so that they can be carried in our pockets in a natural way. Taking advantage of the ubiquity of mobile phones, their sensors, communication and computing capabilities in utilizing context recognition for ITS applications can provide systems that can gather, send and analyze data on-the-fly, recognize activity/context and assist users in an efficient and effortless manner. Accurately recognizing daily activities and detecting transportation modes would allow for several practical applications. These applications include the conducting of more informative and accurate transportation surveys, better urban planning and traffic management, CO_2_ emission contribution and carbon footprint of an individual, more accurate positioning algorithms, travel time estimation and journey planning that account for the vehicle type and context-aware specialized advertisements.

The travel time of different kinds of vehicles can be estimated with better accuracy if the mode of transport can be determined [2]. This is due to the fact that different types of vehicles cover the same distance in varying times. Vehicle classes that cause traffic density can be determined via crowdsourcing techniques [3]. Each vehicle has diverse effects on the road it traverses. Thus, the road conditions can be observed and maintained, if required. Observing how drivers of certain vehicles generally behave and analyzing road conditions [4] would allow the necessary precautions to be taken and improve safety on the roads. Additionally, detecting a general pattern of the movement of vehicles would provide better information about the traffic [5]. Urban and infrastructure planning can be made to account for internal dynamics of the city [6]. For instance, urban planners would have the knowledge of how many people use which transport mode for how long on a daily, monthly or yearly basis. This information would give an idea about the efficiency of current urban planning so that government and emergency services, including ambulances or fire departments, can be deployed more conveniently. Questionnaires/surveys are a way to gather information about transportation systems. However, they are restrictive and prone to inaccuracy as the participants may fill them in long after the initial trip. Furthermore, it is unpractical and deterring for the respondent for a survey to be repeated every time a change needs to be measured. On the other hand, if the survey is conducted via voluntary data gathering, the necessary data can be collected with a few button clicks of the respondent. Thus, a transport mode detection application would allow for easier and more accurate transportation surveys [7]. Conducting transportation surveys can contribute vastly to urban planning.

This paper aims to contribute to smartphone-based activity recognition in ITS applications in three ways:

1. A novel post-processing algorithm, namely Healing, for improving the results after transport mode classification
2. Developing a new set of features for determining the type of movement.
3. Determining the optimum window size and sampling frequency for feature extraction

A key contribution of our work is introducing a novel approach to improve the results after the initial classification process. In this approach, a fixed-size sliding window extracts features for the classification process. Then, the classified data stream is partitioned into segments using “pedestrian state” as a separator. Different from the studies that use pedestrian activities for classification [6,8], we innovatively use this partitioning scheme in order to improve the results by using our post-processing Healing algorithm. In addition to the Healing algorithm, we also introduce a new set of statistical features and demonstrate that these features improve the overall recall rate of transport mode detection by 5%. Previous research had conflicting results as some papers challenged large window sizes for transport mode detection purposes, while others praised it. One of the secondary contributions of the proposed study is the series of analyses conducted for determining the optimum window size, sampling frequency and overlapping ratio.

The remaining sections are given as follows. Section 2 gives a brief history of transport mode detection studies and explains our differences from other studies. In Section 3, we introduce our system architecture based on the proposed Healing algorithm. We give detailed experimental results in Section 4. Section 5 discusses the obtained results and gives future directions. Then, we conclude the paper in Section 6.

## 2. Related Works

The success of the studies about activity recognition has encouraged many researchers to work on transport mode detection [9,10]. Especially to analyze the mobility of a city, several research groups, even R&D departments of Google and Apple, have implemented software for smartphones in order to understand the travel mode of a person. At first, most of the studies have exploited the GPS sensor [2,11,12,13,14,15,16,17,18,19] and accelerometer [6,8,20]. The overall success rate was reported to be 84% in [11], which could detect transport vehicles such as walking, running, cycling, motorcycles, buses and subways by using GPS data. However, due to its high energy consumption, the usage percentage of GPS has decreased in time. On the other hand, sensors such as gyroscopes [2,21,22], microphones, orientation and light sensors are included in addition to accelerometer sensors [2]. The work in [11,21] tried to use the individual sensors both one at a time and together and have observed the importance of the accelerometer sensor with the results obtained. Moreover, a study where a barometer was used for transportation mode detection was also encountered [23]. The work in [24] is one of the most interesting. The authors state in the study that the power consumption of the GPS and other sensors is high, so the voltage values are used. First, classification occurs to differentiate between pedestrian (walking, running) or motorized states (car, train, bus). If the motion was not determined to be walking or running, the system would proceed to determine which type of vehicle was used. However, the study does not employ smartphones; instead, wearable hardware is used. In light of the literature review, we prefer to use the accelerometer, magnetometer and gyroscope, which have proven to be successful in many studies, instead of the GPS sensor because of its high energy consuming structure.

The literature review shows us that walking and being in vehicle states are successfully classified in many studies [2,6,8,11,21,25]. Researchers also investigated the discriminative features [26] for traditional transport modes. They presented the experimental results in two modes of classification: transportation mode (still, walk, run, bike and vehicle) and vehicle mode (high speed rail, metro, bus, car and train) classification based on three machine learning algorithms: Decision Trees (DT), k-Nearest Neighbor (k-NN), and Support Vector Machine (SVM). They noted that classifying vehicle modes is a more difficult task than deciding transportation modes. In [6], cars and trams are classified with a success rate of 82.05%. In [2], less common vehicles, such as two-wheeled motorcycles and three-wheeled tractors, were classified with a success rate of over 90% using the four-fold cross-validation methodology. Different from other studies, [22] was able to identify national buses, as well. Unlike the studies that concentrate on a relatively small number of classes or the studies that distinguish pedestrian activities such as walking, running, still from being in a motorized vehicle [25], in this study, we perform the classification of pedestrian activities and a wide variety of motorized activities including car, bus, tram, train, metro and ferry at the same time.

There are some studies that combine different classification algorithms in order to obtain high success rates. In [2], the researchers built a special case of a committee of learners, which includes DT, k-NN, HMM, SVM and naive Bayes classifiers and made the final decision by majority voting. In [22], the researchers combine multiple learners through cascading in order to increase the overall accuracy by using the set of learners ordered on the basis of their computational costs. They achieved an overall success rate of 88% in classification of still, walk, car, train, bike, city bus and national bus by using time domain features derived from accelerometer and gyroscope data.

In [8], the researchers rely on a three-stage (kinematic motion, stationary and motorized) hierarchical classification framework for transportation mode detection. They combine an instance-based classifier with the discrete hidden Markov model for the kinematic motion classifier. The stationary and the motorized classifiers, on the other hand, perform segment-based classification where a simple voting scheme is used to aggregate frame-based classifications, which are obtained using an instance-based classifier over the observed segment. Pedestrian activity is detected with a success rate of 99%, whereas stationary and motorized states are recognized with 95% and 80%, respectively. A more accurate calculation of gravitational components is stated to be a novel contribution of the paper, as well as breaking down the classification task hierarchically. In our study, instead of combining multiple classifiers, we combine a two-stage thresholding mechanism with well-established machine learning methods in order to achieve high success rates with lower computational costs. Similar to [8], adapting segment-based feature extraction is also encountered in [6]. The authors employ a system where walking activities are detected first, and the interval between walking activities is considered to be a segment in order to perform feature extraction on these segments. In order to detect walking activity, the authors calculate the magnitude of accelerometer data and compare it to a specified threshold. Between two walking activities, vehicle recognition is performed. The key difference of our work from the studies that rely on segment-based classification is that we perform a window-based feature extraction and exploit walking as a separator in order to repair the misclassified windows within the corresponding segment.

On the other hand, there are studies applying a post-processing technique, namely Discrete Hidden Markov Model (DHMM) on accelerometer data. In [27], the researchers assume that some transitions between transportation modes may happen more or less frequently and use Hidden Markov Model (HMM) as a natural way of modeling the temporal evolution of the transportation modes. Their DHMM-based post-processing provides an overall improvement of recall values on average by 2%. Similarly, the researchers combined decision trees with DHMM in [14,28]. Reddy et al. observed an improvement of 2% in recall by exploiting DHMM in [14]. Although applying DHMM as a post-processing smoothing method is a wise approach, the achieved improvement ratios are below expectations. The working principle of DHMM-based smoothing is finding the most probable sequence of states within a particular sequence of observations with the help of the Viterbi algorithm. However, this approach brings the risk of a route-dependent learning. The main differences of our proposed Healing algorithm are its efficiency and route independent structure with high improvement  rates.

To the best of our knowledge, most of the existing studies mainly focused on the performance of classification algorithms, the selection of discriminative features for transport mode detection [20,22,29,30] and fine tuning of parameters including window size, sampling frequency and size of training data [31,32,33,34]. Although machine learning algorithms such as J48 [21,35], SVM [21,36], Sequential Minimal Optimization (SMO) [35], Naive Bayes [21,36] and k-NN [36] give satisfactory results, the best results were obtained with the random forest algorithm as given in [22,36,37]. In [21], the frequency of data gathering is selected as 10 Hz. For feature extraction, a 10-s window size with no overlap has been used. On the other hand, the same as our results, the authors in [38] claim that a 60-s window is one of the optimal parameters for transport mode detection. In [36], time and frequency domain features are extracted with a 20-s window size. In [2], the data are read from the sensors with a frequency of 18 Hz. Time domain features such as minimum, maximum and variance, frequency domain features including energy and Fast Fourier Transform (FFT) results are extracted. Shafique et al. [37], on the other hand, have succeeded in increasing the success rate by choosing a larger window size, as opposed to other studies. In [12], a classification result is obtained using 5-min periods of operation. During the classification process, the authors first determine whether the current activity is either walking, stationary or motorized vehicle, and then, if the activity is indicated as motorized vehicle, the classification algorithm aims at detecting the type of vehicle. In [35], it is noteworthy to point out that the frequency is 33.33 Hz. A few studies [39,40] have addressed the battery-efficiency issue.

In this study, in addition to time domain features, which have a proven success in the area of activity recognition and transport mode detection, a new set of statistical features regarding the distribution of the data is also presented. The contribution of the proposed new set of features and the effect of sampling frequency, window size, overlapping ratio and number of features on the classification success are discussed.

Although there are various applications developed by using many different feature extraction methods and classification approaches, there are a few attempts to correct the classification results by using the transitional nature of transport modes [14,27,28]. In this study, we propose a novel Healing algorithm that can be applied to any transport mode classification scheme in order to improve classification results in a route-independent manner.

Another deficiency of the studies in the literature is the difficulty of comparing the success of existing approaches, since quantitative results are strongly related to the database used and nearly all existing studies collect their own databases to compute their success rates. There are a few studies that use the HTC transportation mode dataset in order to compare their results [25,26]. In this study, we present the classification results of our dataset, which includes a wide variety of vehicular and pedestrian activities including walking, still, bus, car, ferry, metro, train and tram, as well as the results obtained from the HTC dataset in order to compare the success of our proposed algorithms to the state-of-the art.

## 3. System Architecture

**Figure 1:**
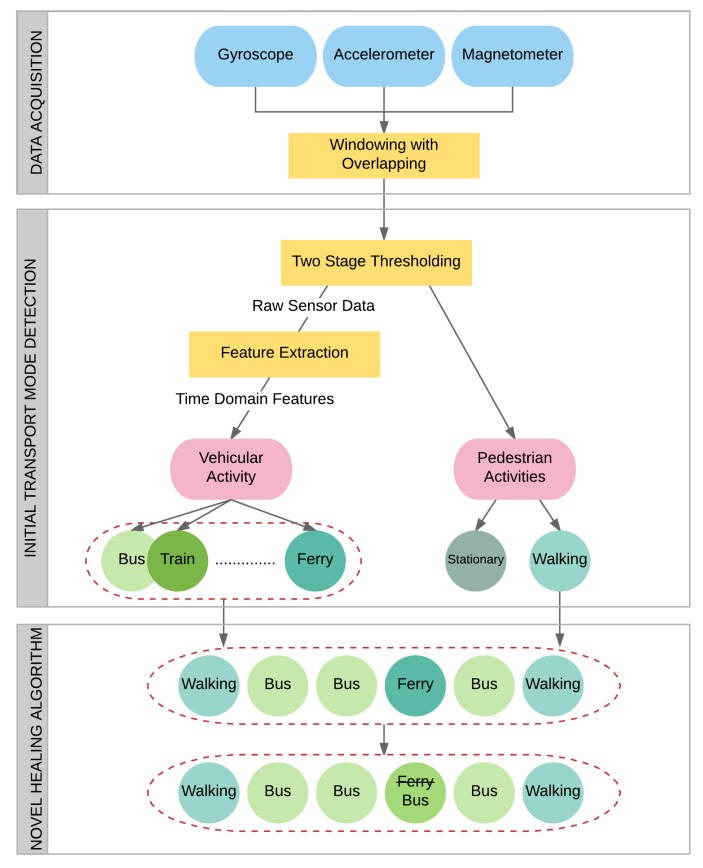
Multi-tiered architecture for transport mode detection.

In this study, we introduce a novel, multi-tiered architecture, which relies on the accelerometer, gyroscope and magnetometer sensors of a smartphone for the purpose of transport mode detection. Our multi-tier architecture is composed of *Data Acquisition*, *Initial Transport Mode Detection* and *Healing Algorithm* steps as shown in Figure 1. After gathering raw sensor data, a set of thresholds is applied in order to detect vehicular activity and to distinguish Stationary and Walking activities. Classification of vehicular activities is then performed by exploiting time domain features with the help of machine learning techniques. In the last step, a novel post-processing algorithm, which is proposed for healing the classification results, is employed.

Improving the initial classification results after a journey is the main contribution of this study. For this purpose, walking activities are used as separators. Exploiting walking activity as a separator between other activities was already discussed in a few papers [6,8]. In these studies, features are extracted based on segments, which refers to the classification of the entire segment at once. Our approach differs from state-of-the-art methods as we perform initial transport mode detection using a window-based approach. We first classify the data using the features extracted from windows. Then, we determine the most often occurring activity between walking segments. Correction of each segment is done by a majority vote.

### 3.1. Data Acquisition

We have collected a total of 79 h of data involving eight participants with different genders and ages in the range of 20 to 45 years old. We developed an Android smartphone application for acquisition of the data and for obtaining the ground-truth information. The diversity of transportation means in Istanbul is rather extensive; featuring four types of buses (bus, metrobus and two different types of minibus), five types of railway vehicles (tram, train (Marmaray), metro and light-rail), ferries and car. The dataset consists of trips with different routes and lengths. The number of trips and the amount of data instances recorded for each mode are given in Table 1.

Data from the accelerometer, gyroscope and magnetometer sensors of the smartphone are gathered at a frequency of 100 Hz. This allows us to downscale the frequency in case of necessity. These sensors record data in three dimensions (e.g., accelerations along *x*-, *y*- and *z*-axes). In addition to the data recorded along three axes, signal vector magnitude values of each sensor are obtained individually by using Equation (1) resulting in a total of 12 inputs to perform feature extraction.

(1) $$Accmag=\sqrt{(}accx^{2}+accy^{2}+accz^{2})$$

According to our observations during dataset collection, each transportation mode takes at least 1  min including walking and stationary actions. Therefore, the acquired data were evaluated within 60-s windows using 40% overlapping in order not to misclassify activities, especially at a transition period between two activities.

### 3.2. Initial Transport Mode Detection

Initial transport mode detection is accomplished by employing Vehicular Activity Detector and Vehicular Activity Classifier components. The Vehicular Activity Detector determines whether a vehicular, stationary or walking activity occurred in the current window. The Vehicular Activity Classifier, on the other hand, decides on the type of vehicle used for transportation and functions in case any stationary or walking activity is not detected.

#### 3.2.1. Vehicular Activity Detection

The Vehicular Activity Detection algorithm, which distinguishes walking, stationary and vehicular activities, is based on a two-stage thresholding approach. Preemptively detecting such actions avoids classification overhead and minimizes classification errors.

In the first stage, the standard deviation of the gathered accelerometer magnitudes of each second, which is denoted by *AccMag*, is calculated, and the transport mode of the corresponding second $Sec_{TM}$ is expressed by Equation (2) where “*St*” and “*W*” are abbreviations for stationary and walking, respectively:

(2) $$Sec_{TM}=\left\{\begin{array}{cc} “W” & if\;stddev\left(AccMag\right)\geq Th_{W}\\ “St” & if\;stddev\left(AccMag\right)\leq Th_{St} \end{array}\right\}$$

**Figure 2:**
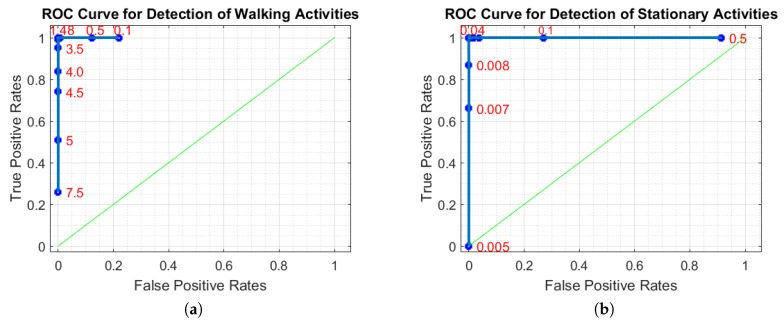
ROC of (**a**) walking activity detection by varying the values of $Th_{W}$ and (**b**) stationary activity detection by varying the values of $Th_{St}$

Since the thresholding parameters $Th_{W}$ and $Th_{St}$ are crucial in the success of stationary and walking activities detection, they were determined using a validation set consisting of 24 trips. Figure 2a,b shows the ROC (Receiver Operating Characteristic) curve of stationary and walking activities detection by varying the values of $Th_{W}$ and $Th_{St}$, respectively. We determined threshold values as 1.48 and 0.04, which produce the minimum number of false positives while maximizing the detection rates of corresponding activities.

The second stage of thresholding detects whether the user is performing a non-vehicular activity by processing the entire window according to Equation (3). Since encountering a vehicular activity that is shorter than 30 s is nearly not possible, if the total number of walking and stationary seconds within a minute is more than 30, than that window is labeled as the most frequent one of either.

(3) $$TM_{win}=\left\{\begin{array}{cc} Argmax_{tm}\left(\sum Sec_{tm=“W”},\sum Sec_{tm=“St”}\right) & if\;\left(\sum Sec_{tm=“W”}+\sum Sec_{tm=“St”}\right)>30\\ “\mathrm{Vehicular}” & \mathrm{otherwise}; \end{array}\right\}$$

If no stationary or walking states were detected, vehicular activity classification commences.

#### 3.2.2. Vehicular Activity Classification

The type of vehicle used for transportation is determined by the Vehicular Activity Classifier. Classification of vehicular activities is composed of two parts: feature extraction and classification.

A total of 12 values including three axes and the signal vector magnitude of these three axes is calculated for each sensor. In total, 29 time domain features consisting of 17 traditional and 12 new proposed features are extracted from the raw data obtained via accelerometer, gyroscope and magnetometer. A total of 348 features is calculated by processing the windows of 60 s. Extracted time domain features are demonstrated in Table 2 where Sensor Data (SD) corresponds to the set of sensor readings within the current window of size N and $sd_{i}$ corresponds to the i-th value of that window.

In addition to the first 17 time domain features, which have a proven success in the area of activity and transport mode detection [41], in this study, we introduced a new set of features in order to have a better understanding of the movement of a vehicle, such as the total amount of times that the vehicle accelerates, decelerates and remains at a constant speed. To achieve this, statistics regarding the distribution of the data within a determined range is exploited. At the first step, a baseline value (BaseLine) and an epsilon (ϵ) is specified. The distribution of the values that lie between and out of the boundaries (BaseLine−ϵ to BaseLine+ϵ) in a window is considered. The total number of values that are above the upper bound, below the lower bound and between the boundaries indicates the features Freq_above_median, Freq_below_median and Freq_between_median, respectively, where BaseLine is specified as the median of the data within the current window. Freq_above_mean, Freq_below_mean and Freq_between_mean are calculated the same way where BaseLine corresponds to the mean of the data. The remaining six features give a clue about the continuity of the data. MaxConsecutive_above_median indicates the maximum number of the values that are consecutively above the upper bound, whereas the maximum number of the values that are consecutively below the lower bound and between the boundaries corresponds to MaxConsecutive_below_median and MaxConsecutive_between_median where BaseLine is specified as the median. Likewise, MaxConsecutive_above_mean, MaxConsecutive_below_mean and MaxConsecutive_between_mean are computed by setting the BaseLine to the mean of the data.

Classification of vehicular activities with high accuracy plays a crucial role in building a robust model. We considered four different supervised learning approaches for classification: k-NN, which realizes instance-based learning, naive Bayes, which is a probabilistic learning model, and finally, random forest and J48, which are decision tree-based methods. A total of 2939 min of sensor data, which belong to the first 158 trips of the collected dataset, is used as the training and validation set in order to compare the performances of the algorithms and to adjust the classification parameters. Weka software was used to perform the tests [42]. Table 3 demonstrates the average recall values of the classification algorithms.

### 3.3. Improvement of Classification Results by the Proposed Healing Algorithm

The primary contribution of this study is the novel segment-based post-processing algorithm, which is designed to improve the classification results. Once a trip has been completely classified using the Vehicular Activity Detection and Vehicular Activity Classification modules, the results undergo a so-called “Healing” process. Healing is a segment-based approach, unlike the window-based initial classification process. To heal the results, the most crucial step is to determine the walking sequences. The underlying idea behind the design of the proposed Healing algorithm is exploiting the absolute existence of walking between two transportation mode changes. It would almost be impossible to, e.g., leave a metro and take a bus without having walked some distance first. This also leads to the conclusion that between two walking events, only one unique transportation activity can occur. At its simplest definition, the proposed algorithm determines that the most activity occurred between two walking events and labels the whole segment as the corresponding activity. The pseudo-code of the algorithm is given in Algorithm 1.

To be able to improve the robustness of the system, we have fine-tuned the proposed algorithm to cover three special cases encountered in daily life.

**Figure 3:**
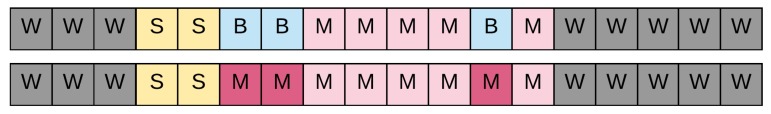
Results of initial classification (first row) and the Healing algorithm (second row) for the first special case.

The first of these cases is the occurrence of a stationary period right after or just before a walking sequence such as waiting for the metro after walking to the station. Since the time spent waiting for a transportation vehicle should not be considered as time spent traveling by that vehicle, the mentioned stationary period will be preserved instead of being corrected by the Healing algorithm. Classification results obtained for the following travel sequence are given in the first row of Figure 3. The user first walks for a minute or more, remains stationary for 2 min, then proceeds to use the metro. After traveling with the metro for 8 min, the user leaves the metro and starts walking again. The classification results obtained by processing the Healing algorithm are given in the second row. The stationary period after the first walking event is ignored. Then, the remaining classes are counted. Since there are three bus and five metro instances in the segment, the whole segment is corrected as metro travel for 8 min.

**Algorithm 1** Healing algorithm.1:i←02:numberOfStationary←03:count[vehicleType]←04:**while**$activity\left[i\right]\neq^{\prime }walking^{\prime }$ **do**5:  **while** activity[i]=’vehicular activity’ **do**6:   count[vehicleType]++7:   *i*++8:    j←i9:    **while** $activity\left[i\right]{=}^{\prime }stationary^{\prime }$ **do**10:        numberOfStationary++11:        *i*++12:    **if** numberOfStationary>=30 **then**13:        k←j14:        **while** $activity\left[k\right]\neq^{\prime }walking^{\prime }$ **do**15:             $activity\left[k\right]{=}^{\prime }stationary^{\prime }$16:             *k*++17:    **if** numberOfStationary>=5 OR $activity\left[i+1\right]{=}^{\prime }walking^{\prime }$ **then**18:         **for** (i=0; i<numberOfActivity; i++) **do**19:        activity[i]←typeOf(max(count[activityType])

**Figure 4:**
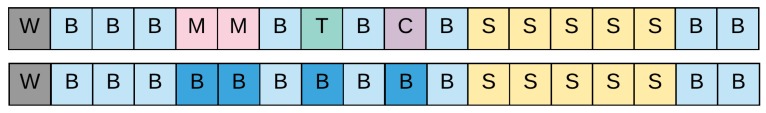
Results of initial classification (first row) and the Healing algorithm (second row) for the second special case.

The second special case is the presence of a stationary period that lasts at least 5 min within travel. It is possible that there may be short stationary sequences during vehicular activities, e.g., waiting for a traffic light. However, if these waiting periods are long, such as traffic congestion due to a road accident, it cannot be concluded that the user is moving during that time. Therefore, just like the first case, the mentioned stationary period will be preserved instead of being corrected by the Healing algorithm. An example of this case could be given as follows: The user walks and takes a bus. After traveling by bus for ten minutes, the user leaves the bus and starts waiting for another bus for 5 min. The journey continues by the second bus. The obtained initial classification results and the results of the Healing algorithm are demonstrated in the first and second rows of Figure 4, respectively. As the most detected transport mode, the algorithm labels the segment as bus, preserving the stationary period.

**Figure 5:**
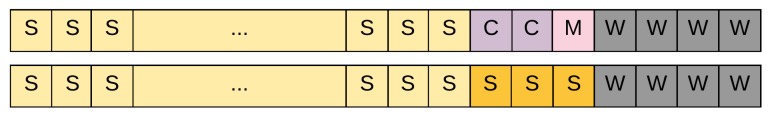
Results of initial classification (first row) and the Healing algorithm (second row) for the third special case.

The last case includes the misclassified activities, which follow a long period of a stationary event, e.g., sleeping. Classification results obtained for the following activity sequence are given in the first row of Figure 5. The user wakes up, uses his/her smartphone for some time and starts walking. In order to correct the misclassified segments, stationary sequences that are longer than half an hour are treated as separators between activities like walking. Therefore, the activity segments that start with a stationary period, which is more than half an hour and terminates by walking, are labeled as stationary (second row of Figure 5).

## 4. Experimental Results

We evaluated the performance of the proposed method in three different sections. In the first section, we have examined the change in success rates of the initial transport mode detection with data acquisition parameters and the number of features. Section 2 evaluates the performance of the multi-tiered initial transport mode detection architecture. Finally, the strength of the proposed Healing algorithm to improve the classification results is revealed in the Section 6. In order to tune and examine the effects of the system parameters, three-fold cross-validation has been applied to 70% of the collected data. The performance of the initial transport mode detection and proposed Healing algorithm is evaluated by the remaining 30% of the dataset. The performance metrics used for evaluation include precision and recall values, which are calculated by Equations (4) and (5), where TP represents the number of correctly labeled instances and FP and FN correspond to the number of false positive and false negative samples, respectively.

(4) Precision=TP/(TP+FP)

(5) Recall=TP/(TP+FN)

### 4.1. Effects of Data Acquisition and Feature Extraction Parameters on Transport Mode Detection Performance

**Figure 6:**
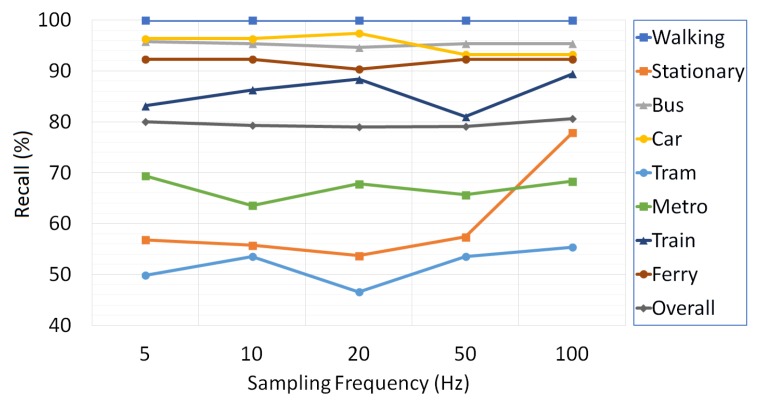
The effect of sampling frequency on the classification results.

**Figure 7:**
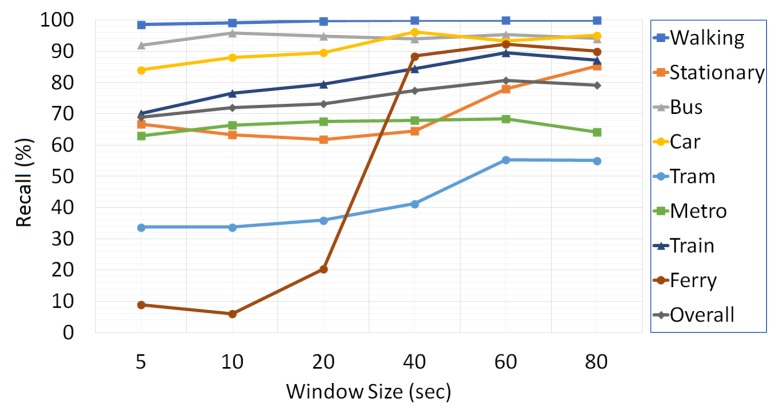
The effect of window size on the classification results.

**Figure 8:**
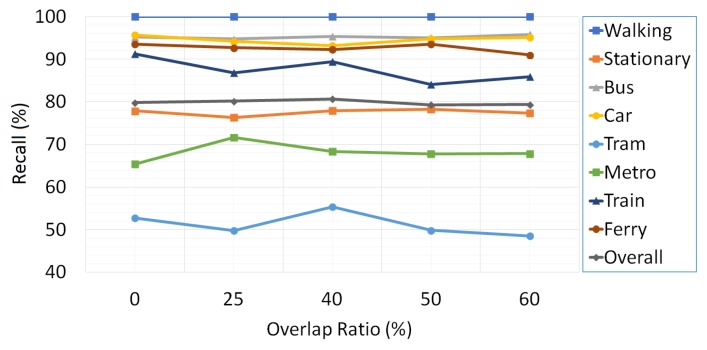
The effect of the overlapping ratio on the classification results.

The effects of three basic data acquisition parameters including sampling frequency, overlapping ratio and window size on the performance of the transport mode detection are examined. With the intent of finding out the significance of data acquisition parameters in the success of the proposed multi-tier architecture, we take advantage of the collected training and validation set. The change in the classification success of transport modes with varying sampling frequency rates, overlapping ratio and window size is given in Figure 6, Figure 7 and Figure 8, respectively.

It is observed that 100% or very close success rates of walking state detection are obtained in all varying data acquisition parameters. These results indicate the robustness of the proposed thresholding-based walking state detection mechanism against the sampling frequency, overlapping ratio and window size parameters. The stationary state involves standing still and the small movements that do not change the location of the subject. Therefore, instantaneous changes in the body position affect the classification success and cause relatively low recall rates.

As shown in Figure 6, the sampling frequency rate does not seem to have much effect on overall system performance, but yet, the highest stationary action recall rate is obtained with a sampling frequency rate of 100 Hz. Detection of stationary and walking activities with high success is crucial since they are treated as separators by the proposed Healing algorithm. This fact leads us to sample sensor data at 100 Hz.

The effect of varying the values of the window size on the classification results is given in Figure 7. During the analysis, it is observed that the recall rates increase proportionally to the window size in all transport modes, and the highest overall recall rate is obtained by a window size of 60 s. For the greater window sizes, the success rate starts to decline. Therefore, a window size of 60 s is used in the development of the mobile transport mode detection system.

Figure 8 demonstrates the effect of overlapping ratio. Adjusting the overlapping ratio enables correct classification of activities that are in a transition period with a reasonable time complexity. We determine the overlapping ratio as 40% since it provides the highest overall recall rate.

The discriminative features always increase the success rate for the classification problems. In this study, in addition to the common time domain features, we propose a set of new features that essentially represent the statistics regarding the distribution of the data within a determined range. The effectiveness of these features is evaluated by performing the tests involving only common time domain features and the whole feature set. The average recall rates are given in Table 4.

The obtained results show that the proposed new feature set gives better results in all classes except the car class and provide 4.6% overall improvement of system performance.

**Figure 9:**
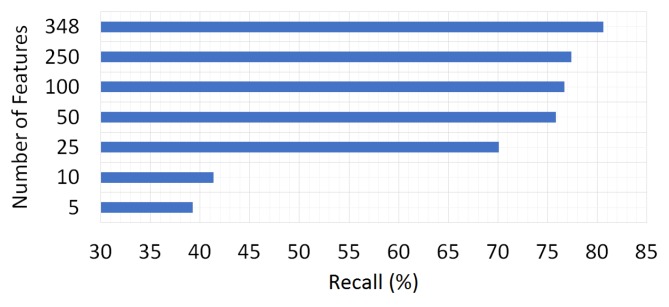
The effect of the number of features on the classification results.

We investigate the possible contribution of the feature selection methods to the performance of the system by exploiting InfoGainAttributeEval algorithm for reducing the number of features from 348 to five. The obtained average recall rates of the system for a varying number of features is demonstrated in Figure 9.

As shown in Figure 9, the highest success rates are obtained by using the whole 348 features. As feature reduction decreases the system performance, we use the whole feature set in order to perform transport mode detection.

### 4.2. Performance Evaluation of the Initial Transport Mode Detection

Vehicular activity detection and vehicular activity classification steps are evaluated separately in order to reveal their effects on the overall system performance.

#### 4.2.1. Evaluation of Vehicular Activity Detection

Detection of vehicular activities and distinguishing walking and stationary conditions with high accuracy is crucial in the success of Transport Mode Detection. The performance of the Vehicular Activity Detection is evaluated by using the validation data. The confusion matrix that is obtained by classification of pedestrian and vehicular activities is given in Table 5.

The proposed two-stage thresholding mechanism used for the detection of vehicular activities also distinguishes between stationary conditions and walking activity, which is directly related to the success of the Healing algorithm. It is observed that high recall rates of 100% and 76% are obtained in identifying walking and stationary sequences, respectively, which also yields good transport mode classification results.

#### 4.2.2. Evaluation of Vehicular Activity Classification

**Figure 10:**
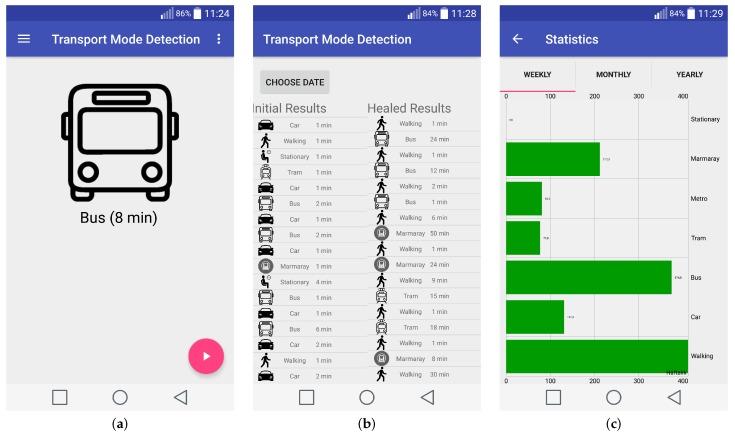
User interface of mobile Transport Mode Detection application. (**a**) Transport Mode Detection Screen; (**b**) results of Initial Transport Mode Detection and Healing algorithm for a given date; (**c**) Statistics of user actions.

In order to realize the proposed multi-tiered transport mode detection, a mobile application for Android smartphones is implemented. The user interface of the transport mode detection application is composed of three major components: real-time transport mode detection, applying the Healing algorithm to the initial transport mode detection results and displaying transport mode statistics of the user. The screen shots of these components are given in Figure 10a–c, respectively.

The performance of vehicular activity classification is evaluated by real-time tests, which are conducted by running the mobile application on two different smartphones; Samsung Galaxy S4 and LG G3, which operate Android 5.0.1 and Android 5.0 Lollipop, respectively. The confusion matrix obtained by employing the Vehicular Activity Classifier is given in Table 6.

### 4.3. Performance Evaluation of the Proposed Healing Algorithm

The proposed Healing algorithm is invoked at the end of the trip. The confusion matrix calculated by the Healing algorithm is proposed in Table 7.

**Figure 11:**
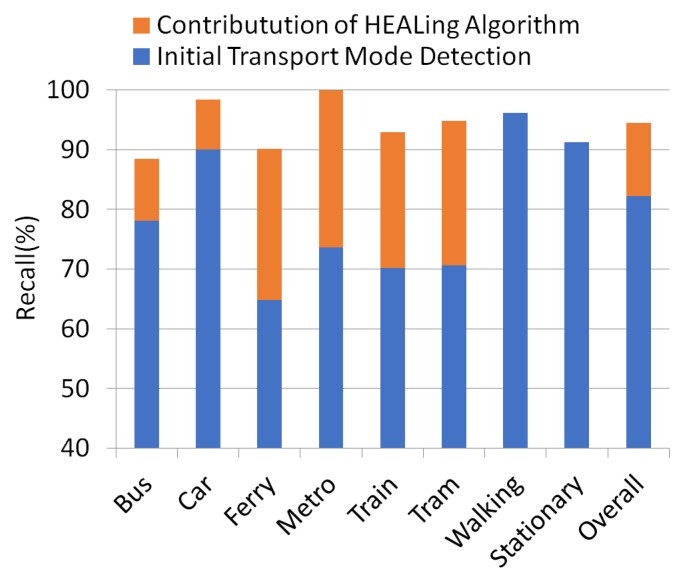
Contribution of the Healing algorithm to the recall of initial transport mode detection.

The results indicate that the newly-proposed Healing algorithm increases the overall system success by 15 points. Calculated recall values of each transport mode with the initial transport mode classification algorithm and the results after the Healing algorithm is employed are demonstrated in Figure 11.

### 4.4. Performance Comparison to the State-Of-The-Art

To the best of our knowledge, most studies use their private datasets collected in major cities including Kobe, Melbourne and Zurich for their experimental results. The only public dataset that is partially available and accessible was collected by HTC company [25]. Thus, to show the success of our system architecture and the Healing algorithm, we ran several tests on this public dataset. A few studies [25,26,43] have already evaluated their methods using this dataset. However, classification of vehicle types is covered by only one of these studies [26]. Other ones focused on distinguishing non-motorized activities from being in a vehicle. Table 8 demonstrates the weighted average recall values of our proposed method before and after applying the Healing process and the performance of the system proposed by Fang et al. [26]. Since our proposed Healing algorithm makes a trip-based post-processing, differently from the system proposed by Fang et al., we also include walk and stationary classes in the classification task. Regarding the success rates shown in Table 8, we can safely claim that our transport mode detection approach outperforms the latest studies using the HTC dataset by up to six points.

On the other hand, the close success rates of our method before applying the Healing algorithm and the system proposed by Fang et al. reveal the opportunity to improve the results of various transport mode detection systems by using the proposed Healing algorithm.

## 5. Discussion

Transport mode detection is an emerging research problem for urban planning in smart cities and intelligent transportation systems. Most studies about transport mode classification investigated the performance of machine learning algorithms. Some of them also evaluated the effect of window size, sampling frequency and the size of training data on the success rate of transport mode detection. The researchers also draw attention to the type of sensors used for this problem. One group pointed out the importance of GPS, whereas other researches avoid GPS and exploit the accelerometer, gyroscope, magnetometer and barometer due to their low energy consumption. In contrast to other studies, we propose a multi-tier architecture for transport mode detection consisting of thresholding, machine learning and a novel post-processing algorithm. Thanks to our post-processing algorithm, namely Healing, we could achieve up to a 94.5% overall recall rate of transport mode identification in real-life scenarios. The Healing algorithm is effective at substantially improving the classification results of the vehicular activities. The best improvement is obtained for the ferry instances. The recall rate of ferry is increased from 64.8%–90.1%, which means an improvement about 40%. On the other hand, recognition of pedestrian activities including walking and stationary modes is decided by the thresholding mechanism. Our experimental results show that stationary modes are barely affected by Healing algorithm in a negative way. In particular, the use of mobile phones while sitting and standing causes false prediction for the recognition of stationary modes. Thus, the Healing algorithm is not able to fix such cases, and sometimes, it can distort correct classification results. However, our observations show us that the high recognition ratio of our separator activity, walking, enables the robustness of the entire system.

Our dataset contains a wide variety of transport means compared to other studies. In this study, we have collected a 79-h dataset from 12 different means of transport including minibus, midi-bus, bus, metrobus (runs on a dedicated line), three different metro lines, train (called Marmaray in Istanbul), tram, ferry, car and taxi. The results of our real-life tests demonstrate that the proposed system architecture is considerably robust against the weight of a vehicle, the driving style, the role in a vehicle (passenger or driver) and the road conditions.

To the best of our knowledge, how the orientation/position of mobile phones affects the results of transport mode classification has not been thoroughly researched. During our tests, subjects carried the phone always on the body (rear/front trousers pocket and jacket pocket) in different positions. The subjects also made their journeys in different modes, such as on foot, sitting, forward and in multiple directions including forward, backward and sideways.

We also believe that in the near future, smartphones will be replaced by smartwatches to track the activities of people. Since we sometimes leave our phones on the table, desk, couch, bed, car, bag, etc., smartwatches are the most appropriate candidates for analyzing the daily activities of people. Therefore, as future work, we are planning to implement this multi-tier architecture on smartwatches.

## 6. Conclusions

Transport mode detection has a crucial role in solving traffic problems, especially in big cities. This study aims to increase the detection rate of different travel modes. The proposed multi-tier architecture exploits thresholding, machine learning solutions and the proposed post-processing algorithm. The thresholding approach helped us to decrease the computational complexity, whereas the novel segment-based Healing algorithm improved the average precision and recall rates by 11.7 and 12.2 points, respectively. This paper introduces a new set of time domain features, which infers statistics of the distribution of the sensor data within a determined range. In this study, walking and stationary modes for special cases are evaluated as separators between vehicle rides. Our experimental results show that the high success rate for the detection of walking ensures the robustness of the proposed architecture. We also show that the random forest algorithm is the most successful one in vehicular activity detection among others, as stated in several studies. We also present that the ideal window size is 60 s for transport mode detection, which is still discussed among researchers.

## Figures and Tables

**Table 1:** Transport mode dataset.

Transport Mode	Number of Trips	Total Time (min)
Bus	53	1186
Car	27	500
Ferry	15	179
Metro	56	976
Train	34	462
Tram	25	677
Walking	82	413
Stationary	33	315

**Table 2:** Feature set.

Features	
MinimumReduction	$\forall i\in \left\{1,\ldots ,N-1\right\},\;min\left(sd_{i+1}-sd_{i}\right)\;if\;\left(sd_{i+1}-sd_{i}\right)<0$
MaximumReduction	$\forall i\in \left\{1,\ldots ,N-1\right\},\;max\left(sd_{i+1}-sd_{i}\right)\;if\;\left(sd_{i+1}-sd_{i}\right)<0$
MinimumIncrease	$\forall i\in \left\{1,\ldots ,N-1\right\},\;min\left(sd_{i+1}-sd_{i}\right)\;if\;\left(sd_{i+1}-sd_{i}\right)>0$
MaximumIncrease	$\forall i\in \left\{1,\ldots ,N-1\right\},\;max\left(sd_{i+1}-sd_{i}\right)\;if\;\left(sd_{i+1}-sd_{i}\right)<0$
MinimumValue	$\forall i\in \left\{1,\ldots ,N-1\right\},\;min\left(sd_{i}\right)$
MaximumValue	$\forall i\in \left\{1,\ldots ,N-1\right\},\;max\left(sd_{i}\right)$
Range	MaximumValue−MinimumValue
ArithmeticMean	$(\sum_{n=1}^{N}sd_{i})/N$
HarmonicMean	$N/\sum_{n=1}^{N}\left(1/sd_{i}\right)$
QuadraticMean	$\sqrt{\sum_{n=1}^{N}sd_{i}^{2}/N}$
Mod	$\forall i\in \left\{1,\ldots ,N\right\},\;Argmax_{sd_{i}}\left(freq\left(sd_{i}\right)\right)$
Median	$\tilde{SD}$
Variance	$\left(\sum_{n=1}^{N}{\left(sd_{i}-ArithmeticMean\right)}^{2}\right)/\left(N-1\right)$
StandardDeviation	$\sqrt{Variance}$
Arithmetic Mean of Instant Exchange	$(\sum_{n=1}^{N}\left(sd_{i+1}-sd_{i}\right))/N$
Quadratic Mean of Instant Exchange	$\sqrt{\sum_{n=1}^{N}{\left(sd_{i+1}-sd_{i}\right)}^{2}/N}$
Covariance	StandardDeviation/ArithmeticMean
Freq _above_median	$\forall i\in \left\{1,\ldots ,N\right\},\;freq\left(sd_{i}\right)\;if\;\left(sd_{i}\right)>median+\epsilon$
Freq_below_median	$\forall i\in \left\{1,\ldots ,N\right\},\;freq\left(sd_{i}\right)\;if\;\left(sd_{i}\right)<median-\epsilon$
Freq_between_median	$\forall i\in \left\{1,\ldots ,N\right\},\;freq\left(sd_{i}\right)\;if\;median+\epsilon >sd_{i}>median-\epsilon$
Freq_above_mean	$\forall i\in \left\{1,\ldots ,N\right\},\;freq\left(sd_{i}\right)\;if\;\left(sd_{i}\right)>mean+\epsilon$
Freq_below_mean	$\forall i\in \left\{1,\ldots ,N\right\},\;freq\left(sd_{i}\right)\;if\;\left(sd_{i}\right)<mean-\epsilon$
Freq_between_mean	$\forall i\in \left\{1,\ldots ,N\right\},\;freq\left(sd_{i}\right)\;if\;mean+\epsilon >sd_{i}>mean-\epsilon$
MaxConsecutive_above_median	$\forall i\in \left\{1,\ldots ,N\right\},\;max\left(m\right)\;\;if\;\forall j\in \left\{1,\ldots ,m\right\},\;\left(sd_{i+j}\right)>median+\epsilon$
MaxConsecutive_below_median	$\forall i\in \left\{1,\ldots ,N\right\},\;max\left(m\right)\;\;if\;\forall j\in \left\{1,\ldots ,m\right\},\;\left(sd_{i+j}\right)<median-\epsilon$
MaxConsecutive_between_median	$\forall i\in \left\{1,\ldots ,N\right\},\;max\left(m\right)\;\;if\;\forall j\in \left\{1,\ldots ,m\right\},\;median+\epsilon >\left(sd_{i+j}\right)>median-\epsilon$
MaxConsecutive_above_mean	$\forall i\in \left\{1,\ldots ,N\right\},\;max\left(m\right)\;\;if\;\forall j\in \left\{1,\ldots ,m\right\},\;\left(sd_{i+j}\right)>mean+\epsilon$
MaxConsecutive_below_mean	$\forall i\in \left\{1,\ldots ,N\right\},\;max\left(m\right)\;\;if\;\forall j\in \left\{1,\ldots ,m\right\},\;\left(sd_{i+j}\right)<mean-\epsilon$
MaxConsecutive_between_mean	$\forall i\in \left\{1,\ldots ,N\right\},\;max\left(m\right)\;\;if\;\forall j\in \left\{1,\ldots ,m\right\},\;mean+\epsilon >\left(sd_{i+j}\right)>mean-\epsilon$

**Table 3:** Performance of the classification algorithms on the transportation mode dataset.

Classification Algorithm	Recall
Random Forest	80.62%
J48	72.22%
k-NN	70.04%
Naive Bayes	71.03%

**Table 4:** Performance of the transport mode detection by using only common time domain features and by using the whole feature set.

Transport Mode	Recall Rates Obtained by Using Only Common Features	Recall Rates Obtained by Whole Feature Set
Bus	94.04	95.39
Car	96.87	93.22
Ferry	90.8	92.3
Metro	63.58	68.33
Train	78.94	89.47
Tram	41.02	55.31
Overall	78.17	82.80

**Table 5:** Confusion matrix of vehicular activity detection.

Actual Class	Predicted Class		Ground Truth	Recall
	Pedestrian Activities	Vehicular Activities		
Pedestrian Activities	269	46	315	85.4%
Vehicular Activities	80	2875	2955	97.3%

**Table 6:** Confusion matrix without applying the Healing algorithm.

Actual Class	Predicted Class								Ground Truth	Recall
	Bus	Car	Ferry	Metro	Train	Tram	Walking	Stationary		
Bus	143	12	5	1	6	4	6	6	183	78.1%
Car	6	226	11	1	2	1	4	3	254	90.0%
Ferry	20	2	59	2	0	2	0	6	91	64.8%
Metro	9	4	0	167	1	39	0	7	227	73.6%
Train	22	9	0	8	177	25	0	11	252	70.2%
Tram	8	6	0	19	7	108	0	5	153	70.6%
Walking	6	0	0	1	1	0	331	5	344	96.2%
Stationary	14	9	0	0	0	0	0	242	265	91.3%
**Precision**	62.7%	84.3%	78.6%	83.9%	91.2%	60.3%	97.1%	84.9%		

**Table 7:** Confusion matrix after applying the Healing algorithm.

Actual Class	Predicted Class								Ground Truth	Recall
	Bus	Car	Ferry	Metro	Train	Tram	Walking	Stationary		
Bus	162	15	0	0	0	0	6	0	183	88.5%
Car	0	250	0	0	0	0	3	0	254	98.4%
Ferry	9	0	82	0	0	0	0	0	91	90.1%
Metro	0	0	0	227	0	0	0	0	227	100%
Train	8	0	0	0	234	10	0	0	252	92.9%
Tram	0	0	0	8	0	145	0	0	153	94.8%
Walking	6	5	0	1	1	0	331	0	344	96.2%
Stationary	16	8	0	0	0	0	0	241	265	90.9%
**Precision**	80.6%	89.9%	100%	96.1%	99.6%	93.5%	97.4%	100%		

**Table 8:** Performances of the proposed method and state-of-the-art.

Classification Algorithm	Recall
Fang et al. [26]	83.57%
Before Applying Healing Algorithm	84.38%
After Applying Healing Algorithm	91.63%
